# Organic Cation Transporter 1 an Intestinal Uptake Transporter: Fact or Fiction?

**DOI:** 10.3389/fphar.2021.648388

**Published:** 2021-04-14

**Authors:** Christoph Wenzel, Marek Drozdzik, Stefan Oswald

**Affiliations:** ^1^ Department of Pharmacology, Center of Drug Absorption and Transport, University Medicine Greifswald, Greifswald, Germany; ^2^ Department of Experimental and Clinical Pharmacology, Pomeranian Medical University, Szczecin, Poland; ^3^ Institute of Pharmacology and Toxicology, Rostock University Medical Center, Rostock, Germany

**Keywords:** organic cation transporter 1, intestine, human, gene expression, protein abundance, localization

## Abstract

Intestinal transporter proteins are known to affect the pharmacokinetics and in turn the efficacy and safety of many orally administered drugs in a clinically relevant manner. This knowledge is especially well-established for intestinal ATP-binding cassette transporters such as P-gp and BCRP. In contrast to this, information about intestinal uptake carriers is much more limited although many hydrophilic or ionic drugs are not expected to undergo passive diffusion but probably require specific uptake transporters. A transporter which is controversially discussed with respect to its expression, localization and function in the human intestine is the organic cation transporter 1 (OCT1). This review article provides an up-to-date summary on the available data from expression analysis as well as functional studies *in vitro*, animal findings and clinical observations. The current evidence suggests that OCT1 is expressed in the human intestine in small amounts (on gene and protein levels), while its cellular localization in the apical or basolateral membrane of the enterocytes remains to be finally defined, but functional data point to a secretory function of the transporter at the basolateral membrane. Thus, OCT1 should not be considered as a classical uptake transporter in the intestine but rather as an intestinal elimination pathway for cationic compounds from the systemic circulation.

## Introduction

**FIGURE 1:**
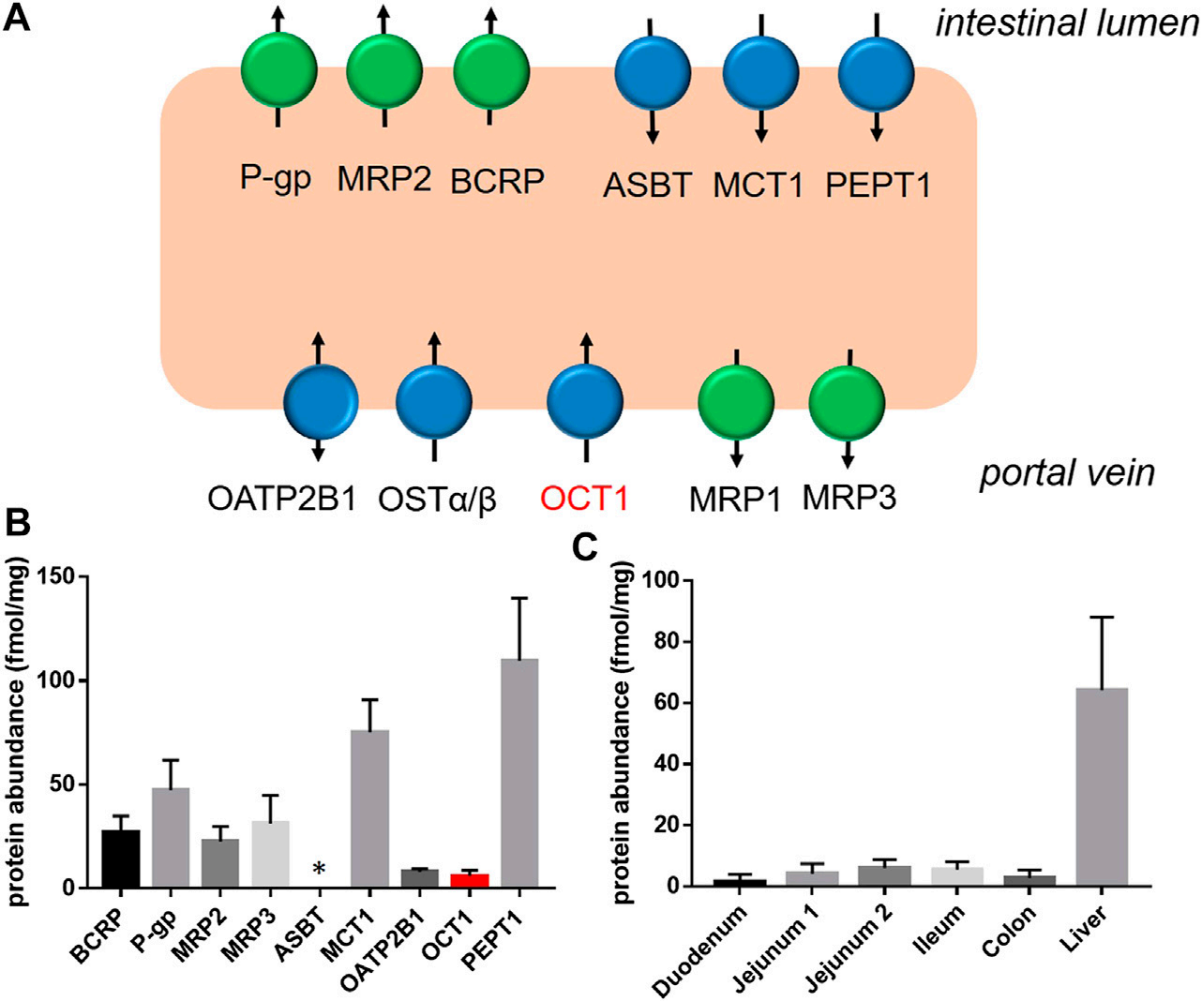
Intestinal drug transporters: **(A)**, Schematic overview of clinically relevant transporters in human enterocytes (blue symbols, SLC transporters; green, ABC transporters); **(B)**, Protein abundance of clinically relevant transporters in the human jejunum, and **(C)**, of OCT1 in different intestinal segments and the liver as observed in nine organ donors using the targeted proteomics approach (Drozdzik et al., 2019).

The intestinal epithelium is by far more than a simple passive diffusion barrier as assumed in earlier days. On the contrary, enterocytes are equipped with many physiologically highly relevant transporter proteins that mediate on the one hand a selective and specific absorption of important nutrients and endogenous compounds including peptides via the peptide transporter (PEPT)1 (SLC15A1), glucose via the sodium dependent glucose transporter 1 (SGLT1, SLC5A1), fatty acids via the monocarboxylate transporter 1 (MCT1, SLC16A1), cholesterol and phytosterols via ABCG5/G8, bile acids via the apical sodium-dependent (ASBT, SLC10A1), and vitamins via the sodium-dependent multivitamin transporter (SMVT, SLC5A6) (Figure 1) (Drozdzik et al., 2014; Estudante et al., 2016; Müller et al., 2017; Drozdzik et al., 2019).

On the other hand, intestinal transporters are recognized as significant determinants of intestinal absorption of many drugs and thus as important factors influencing their efficacy and safety (Giacomini et al., 2010; Hillgren et al., 2013; Zamek-Gliszczynski et al., 2018). In this regard, especially ATP-binding cassette (ABC) transporters such as P-glycoprotein (P-gp, ABCB1), breast cancer resistance protein (BCRP, ABCG2) and the multidrug resistance-associated protein 2 (MRP2, ABCC2) have been extensively investigated. Related to this, inhibition of those transporters resulted in significantly increased absorption of respective transporter substrates (Westphal et al., 2000a; Schwarz et al., 2000; Rengelshausen et al., 2003; Oswald et al., 2006a), whereas induction strikingly reduced their systemic exposure (Greiner et al., 1999; Westphal et al., 2000b; Oswald et al., 2006b). Differences in the longitudinal expression of ABC transporters along the intestine, such as P-gp, were identified as the potential reason for the phenomenon of regio-selective drug absorption (“absorption window”), as observed when comparing different oral dosage forms or by using intestinal perfusion catheter techniques (Gramatté et al., 1996; Weitschies et al., 2005; Tubic et al., 2006; Drozdzik et al., 2014).

In contrast to this, our knowledge is much more limited when it comes to intestinal drug uptake carriers of the SLC family. Although this family contains some 450 members (Hediger et al., 2013), only few SLC transporters have been associated and investigated in terms of their involvement in drug absorption. This is somewhat surprising considering the fact that many drugs are highly polar and permanently or temporarily charged in the rather acidic environment of the upper small intestine (due to their basicity), which are not expected to be absorbed by passive diffusion (Di et al., 2020). Frequently discussed intestinal drug transporters are PEPT1, the organic anion transporting peptides (OATPs) OATP1A2 and OATP2B1 as well as the polyspecific organic cation transporter OCT1. Of the aforementioned carriers, the knowledge on the peptide transporter PEPT1 is the most reliable and consistent. Accordingly, PEPT1 is highly abundant at the apical membrane of human enterocytes along the entire small intestine but not in the colon, and acts as a low affinity-high capacity uptake carrier for peptide-like drugs including beta-lactam antibiotics (e.g., amoxicillin, cefadroxil), angiotensin converting enzyme inhibitors (e.g., enalapril, benazepril) and antiviral drugs (e.g., valacyclovir, valganciclovir) (Brandsch, 2013; Drozdzik et al., 2019). This transporter is even used for innovative drug delivery strategies, in which the oral absorption of several drugs is substantially increased by administering prodrugs being recognized by PEPT1 (e.g., valacyclovir, cefuroxime axetil, oseltamivir) (Kramer, 2011; Brandsch, 2013). OATP1A2 was assumed to be involved in the intestinal absorption of several compounds (e.g., talinolol, nadolol) and responsible for several profound interactions with juices and green tea (Schwarz et al., 2005; Glaeser et al., 2007; Misaka et al., 2014). While gene expression studies were able to detect it along the entire human intestine (Nishimura and Naito, 2005; Glaeser et al., 2007) protein expression could only be verified in one study by immunohistochemistry (Glaeser et al., 2007). In contrast, several more recent studies were not able to detect protein expression by targeted proteomics which leads to the conclusion that OATP1A2 may not be considered as an intestinal transporter (Hilgendorf et al., 2007; Meier et al., 2007; Drozdzik et al., 2014; Miyauchi et al., 2016; Vaessen et al., 2017; Drozdzik et al., 2019). On the contrary, OATP2B1 is homogenously abundant along the human intestine (Drozdzik et al., 2014). As this carrier was shown to be a potent transporter of statins and other drugs *in vitro*, an important *in vivo* role in intestinal drug absorption was hypothesized (Oswald, 2019). However, the enterocyte localization of OATP2B1 remains still uncertain. While Kobayashi et al. found it in the apical membrane using immunohistochemistry analysis (Kobayashi et al., 2003), Keiser et al. via targeted proteomics approach revealed its basolateral membrane expression, which was also confirmed by functional data from vectorial transport studies across human and porcine jejunum in the Ussing chamber (Keiser et al., 2017).

The same controversy exists for the intestinal expression of OCT1, which is predominately (if not exclusively) expressed at the sinusoidal membrane of human hepatocytes where it mediates the hepatic uptake of many drugs (Drozdzik et al., 2019; Hilgendorf et al., 2007; Nishimura and Naito, 2005). In this regard, OCT1 considers especially small (<300–400 Da), hydrophilic and cationic compounds (Table 1). An additional feature of its substrates is, in most cases, a considerable basicity (pKa∼ 8–10) which results in a domination of the positively charged moiety of the drug at physiological conditions (pH 7.4 in the systemic circulation and pH 3–5 in the small intestine). OCT1 was convincingly demonstrated to be involved in the pharmacokinetics of several frequently used drugs as concluded in most cases from *in vitro* and pharmacogenetic studies (Zamek-Gliszczynski et al., 2018). Associated to this, SLC22A1 genetic loss-of-function polymorphisms were associated with diminished hepatic drug uptake, which in turn increased the systemic drug exposure of OCT1 substrates like sumatriptan, fenoterol, tramadol or morphine (Tzvetkov et al., 2011; Tzvetkov et al., 2013; Matthaei et al., 2016; Stamer et al., 2016; Tzvetkov et al., 2018; Matthaei et al., 2019). In this regard, the frequently prescribed antidiabetic drug metformin is almost exclusively eliminated via the kidney which shows expression of OCT2/3 but no OCT1 (Shu et al., 2008; Tzvetkov et al., 2009; Zamek-Gliszczynski et al., 2018). Thus, in contrast to earlier assumptions, the pharmacokinetics of metformin is not expected to be significantly affected by OCT1 (see also chapter: “Evidence from clinical studies”) (Zamek-Gliszczynski et al., 2018). However, conclusions on intestinal OCT1 can only be derived indirectly from those studies. The same is true as for the evidence from clinical drug-drug interaction (DDI) studies in humans. Finally, available *in vitro*, *ex vivo* and *in vivo* models are only partly appropriate to allow conclusive deductions on the function of OCT1 in the human intestine and so far published data have to be interpreted with caution. The following paragraphs will summarize the current knowledge about the expression and localization of intestinal OCT1 as well as available *in vitro*, *ex vivo* and animal study findings. Finally, the evidence from clinical observations will be recapitulated in relation to the potential role of intestinal OCT1 in human drug absorption.

**TABLE 1:** Overview of clinically relevant drugs described to be substrates of human OCT1 and their physicochemical properties as obtained from DrugBank (https://go.drugbank.com). If available, experimental data have been preferred over predicated data (*permanent cations, no pKa available).

Substrate	Drug class	Molecular mass (Da)	logP/pKa	Other transporters/Enzymes involved	Reference
Acyclovir	Antiviral drug	225.2	−1.76/2.5 and 9.4	Alcohol and aldehyde dehydrogenase, OAT1, OAT3, MATE1, MATE2K	Ito et al. (2014)
Amantadine	NMDA receptor antagonist (morbus Parkinson and influenza a drug)	151.2	2.4/10.7	OCT2	Kristufek et al. (2002)
Amiloride	Diuretic	229.6	−0.3/8.7	OCT2, OCTN1	Koepsell (2020)
Amisulpride	Antipsychotic drug	369.5	1.1/9.4		dos Santos Pereira et al. (2014)
Atenolol	ß1-adrenoreceptor blocker	266.3	0.16/9.6	CYP2D6 (minor), OATP1A2	Mimura et al. (2015)
Atropine	Anticholinergic drug	289.4	1.8/9.4		Müller et al. (2005); Koepsell (2020)
Butylscopolamine	Anticholinergic drug, spasmolytic	360.4	−1.9/*		Koepsell (2020)
Cimetidine	Histamine H2 receptor antagonist	252.3	0.4/6.9	FMO1, FMO3, P-gp	Urakami et al. (1998)
Codeine	Analgetic, antitussive drug	299.4	1.4/8.2	CYP2D6, UGT2B4, UGT2B7, P-gp (metabolite)	Meyer et al. (2020)
Diphenhydramine	Histamine H1 receptor antagonist	255.3	3.3/9.0	CYP2D6, CYP2C9, CYP2C19	Müller et al. (2005)
Etilefrine	α-Adrenoceptor agonist (antihypotensive drug)	181.2	0.23/9.7		Jensen et al. (2020)
Fenoterol	ß2-sympathicomimetic, antiasthmatic	303.3	1.4/9.6		Tzvetkov et al. (2018)
Formoterol	ß2-sympathicomimetic, antiasthmatic	344.4	2.2/9.8	CYP2D6, CYP2C9/19, UGTs	Jensen et al. (2020)
Fluoxetine	Serotonin reuptake inhibitor (antidepressant)	309.3	4.1/9.8	CYP2D6, CYP2C9, CYP3A4	Koepsell (2020)
Ipratropium	Anticholinergic drug, bronchospasmolytic	332.5	0.04/*	OCTN1/2	Hendrickx et al. (2013); Chen et al. (2017)
Ketamine	NMDA receptor antagonist (anesthetic)	237.7	3.1/7.5	CYP2B6, CYP3A4, CYP2C9, P-gp	Keiser et al. (2018)
Metformin	Antidiabetic drug	129.2	−2.6/12.4	OCT2, OCT3, MATE1/2 K	Hendrickx et al. (2013)
Metoclopramide	Antiemetic drug	299.8	2.7/9.3	CYP2D6, CYP3A4, P-gp	Hendrickx et al. (2013)
Morphine	Analgetic	285.3	0.9/8.2	UGT2B7, P-gp	Tzvetkov et al. (2013)
Oxaliplatin	Antineoplastic	397.3	−0.5/*	OCT2, OCT3, SLC31A1	Zhang et al. (2006)
Oxybutynin	Anticholinergic drug (overactive bladder)	357.5	4.3/8.0	CYP3A4	Koepsell (2020)
Procainamide	Antiarrhythmic	235.3	0.9/9.3	CYP2D6, OCT2, OCT3, OCTN1/2, MATE1/2 K	Koepsell (2020)
Proguanil	Antimalarial drug	253.7	2.5/10.4	CYP2D6, CYP2C9, CYP2C19	Matthaei et al. (2019)
Ranitidine	Histamine H2 receptor antagonist	314.1	0.2/8.2	CYP1A2, CYP2D6, CYP3A4 (all minor), OCT2, P-gp	Meyer et al. (2017)
Salbutamol	ß2-sympathicomimetic, antiasthmatic	239.3	1.4/10.3		Jensen et al. (2020)
Sulpiride	Antipsychotic drug	341.4	0.6/9.1		dos Santos Pereira et al. (2014)
Sumatriptan	Anti-migraine	295.4	0.9/4.9	MAO-A, OATP1A2, P-gp	Matthaei et al. (2016)
Terazosin	α-Adrenoceptorantagonist	387.2	1.1/7.2	Hepatic CYPs	Hendrickx et al. (2013)
Tiotropium	Anticholinergic drug, bronchospasmolytic	392.5	−1.8/*	CYP2D6, CYP3A4 (all minor), OCTN1/2	Hendrickx et al. (2013)
Triamterene	Diuretic	253.3	1.0/3.1	CYP1A2	Koepsell (2020)
Trimethoprim	Antibiotic	290.3	0.9/7.1	CYP2C9, CYP3A4, CYP1A2	Hendrickx et al. (2013)
Trospium	Anticholinergic drug (overactive bladder)	392.5	−0.5/*	OATP1A2, P-gp	Abebe et al. (2020)

## Evidence From Expression Studies

According to former studies on human OCT1, the transporter was reported to be localized in the basolateral membrane of epithelial cells in kidney, intestine as well as the liver (Jonker et al., 2001; Jonker and Schinkel, 2004; Nies et al., 2009). Thus, it was assumed to be involved in the intestinal excretion, hepatic uptake and renal elimination of endogenous compounds and drugs, although more recent studies have clearly demonstrated that OCT1 was not expressed in the kidney (Prasad et al., 2016; Cheung et al., 2019; Oswald et al., 2019).

**FIGURE 2:**
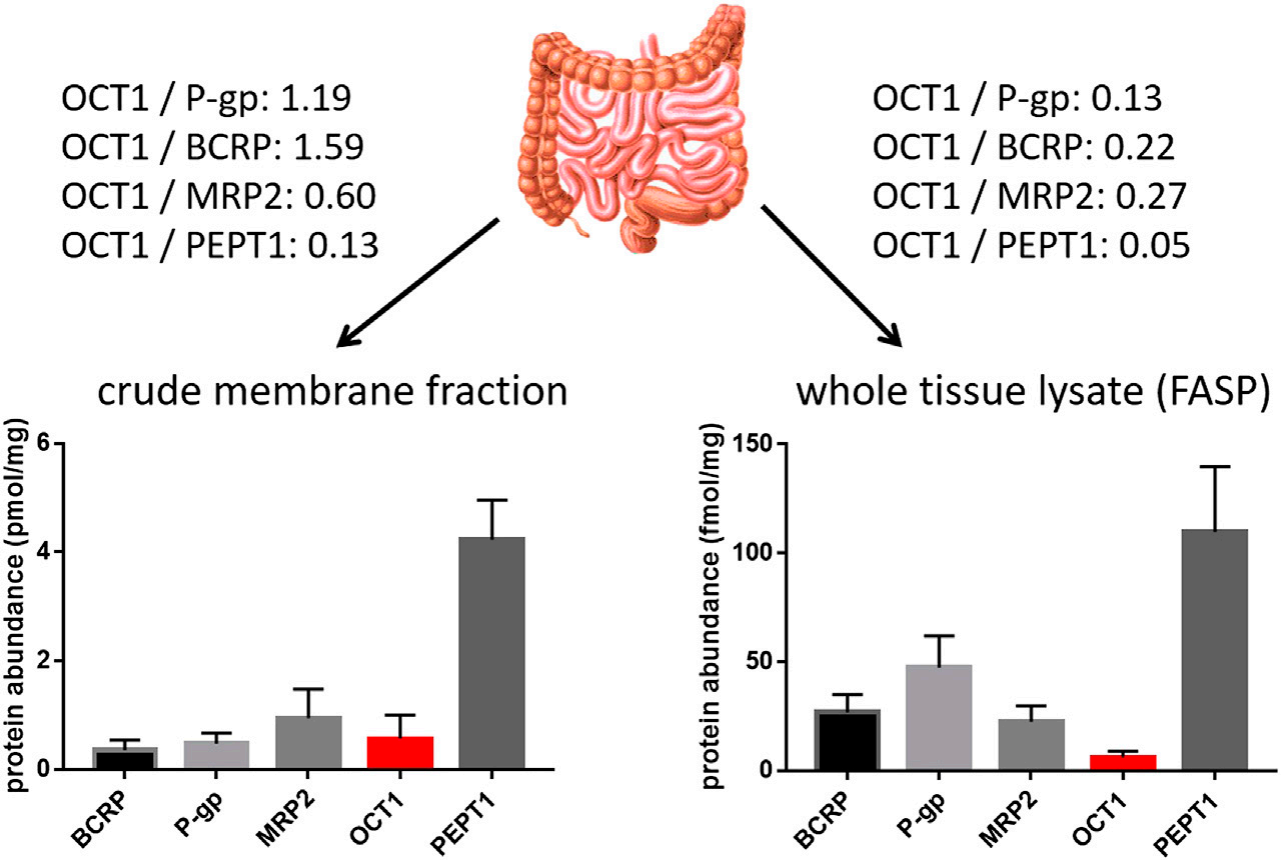
Impact of sample preparation on the observed protein abundance of relevant intestinal transporter proteins in the human jejunum. Data on the left diagram were observed from six organ donors after isolation and targeted proteomics analysis of the crude membrane fration (Drozdzik et al., 2014), while data on the right diagram were observed from nine organ donors after sample preparation using the FASP (filter aided sample preparation) protocol and targeted proteomics analysis of the resulting whole tissue lysates. Relative expression ratios of OCT1 to the other transporters are given.

In contrast to the well-established role of OCT1 in the hepatic disposition of drugs, its role in the intestine remains still unclear. This can be explained by the limited and in part controversial data on its expression there. Several studies unambiguously demonstrated mRNA expression of OCT1 in human intestinal tissue although the expression levels were much lower than that in the liver (Table 2). More recent mass spectrometry-based studies could also verify its protein abundance (Drozdzik et al., 2014; Miyauchi et al., 2016; Vaessen et al., 2017; Zamek-Gliszczynski et al., 2018; Drozdzik et al., 2019). In each case, the protein abundance was low compared to other important intestinal transporters such as P-gp or PEPT1. However, in this regard, the method of sample preparation seems to affect the relative and absolute expression data considerably (Wegler et al., 2017). A comparative analysis revealed that the frequently used protocol of analyzing transporter proteins in the crude membrane fraction may overestimate the amount of intestinal OCT1 compared to whole tissue lysates (Drozdzik et al., 2014; Drozdzik et al., 2019) (Figure 2). Accordingly, analysis of transporter proteins in enriched membranes overestimated the relative expression of OCT1 by 3–9-fold (compared to other transporters). This is most likely due to substantial intracellular sequestration of the transporter and indicates another source of intra-lab variability of targeted proteomics data on drug transporters.

**TABLE 2:** Overview of available data on mRNA expression, protein abundance and localization of OCT1 in the human intestine (+, gene/protein expression was shown; n.d., not detectable; -, not investigated). Data are ranked in chronological order (publication date).

	Small intestine		
References	mRNA	Protein (method)	Localization (method)
Gründemann et al. (1994)	+	−	−
Nishimura and Naito (2005)	+	−	−
Terada et al. (2005)	+	−	−
Müller et al. (2005)	−	+ (immunohistochemsity)	Lateral (immunohistochemsity)
Englund et al. (2006)	+	−	−
Seithel et al. (2006)	+	−	−
Hilgendorf et al. (2007)	+	−	−
Meier et al. (2007)	+	−	−
Han et al. (2013)	−	+ (immunohistochemsity)	Apical (immunohistochemsity)
Gröer et al. (2013)	+	+ (proteomics)	−
Drozdzik et al. (2014)	+	+ (proteomics)	−
Miyauchi et al. (2016)	−	< LLOQ (proteomics)	−
Vaessen et al. (2017)	−	+ (proteomics)	−
Drozdzik et al. (2019)	+	+ (proteomics)	−

With respect to the cellular localization of OCT1, immunohistochemistry analysis by Müller et al. demonstrated lateral localization (Müller et al., 2005). In contrast to this, immunostaining and functional studies by the Thakker lab provided convincing evidence that OCT1 is may be present in the apical membrane of mouse and human enterocytes (Han et al., 2013). In addition, these observations have been also confirmed in Caco-2 cells (see next Chapter). As a consequence of those contradictory findings, recent summaries or review articles have indicated OCT1 either as an apical or basolateral transporter (Estudante et al., 2013; Hillgren et al., 2013; Proctor et al., 2016; Müller et al., 2017). From the functional perspective, this uncertainty cannot answer the question whether OCT1 is directly involved in the intestinal uptake/intestinal absorption (in the case of an apical localization) or rather in the elimination of its substrates from the systemic circulation (in the case of a basolateral localization). In this regard, additional data on the function of OCT1 from *in vitro* and *ex vivo* studies or animal and clinical findings may provide more robust evidence on the expression and function of intestinal OCT1.

## Evidence From Functional Studies *in vitro*, *ex vivo* and Animal Studies

Stably transfected cell lines overexpressing OCT1 (e.g., MDCKII, HEK293 or CHO cells) are well established *in vitro* models for the identification of substrates and inhibitors of OCT1 and other SLC transporters (Tzvetkov et al., 2011; Brouwer et al., 2013; Tzvetkov et al., 2018). However, due to their artificial character, e.g., strong overexpression of OCT1, different background of other transporters, species origin and cell type (different species for MDCKII, CHO and cell type for MDCKII, HEK293 or CHO cells), lack of polarization (HEK293, CHO) and inability to form tight monolayers (HEK293, CHO), those cell models cannot be used for predictive studies on intestinal OCT1 function. A substantial step forward in this direction are Caco-2 cells that originate from human colorectal adenocarcinoma cells. Among advantages of Caco-2 cells is their morphologic resemblance to the intestinal epithelium due to ability of formation of a confluent monolayer of polarized cells with microvilli on the apical side as well as connecting tight junctions (Hidalgo et al., 1989).

Caco-2 cell monolayers have been frequently used to study transepithelial transport of several OCT1 substrates (Lee et al., 2002; Watanabe et al., 2002; Kuwayama et al., 2008; Proctor et al., 2008; Horie et al., 2011; Elsby et al., 2017). Considering their polarity and biorelevant localization of intestinal transporters, studies on the vectorial basolateral-to-apical (B-A) compared to the apical-to-basolateral (A-B) transport in the absence and presence of OCT inhibitors allow assumptions on the cellular localization of intestinal OCT1. Associated to this, a higher B-A (i.e., secretory transport) compared to the opposite direction was observed for sulpiride, ranitidine, famotidine and 3,4-methylenedioxymethamphetamine indicating basolateral OCT1 working in concert with apical P-gp which considers most OCT1 substrates (Lee et al., 2002; Watanabe et al., 2002; Kuwayama et al., 2008). For metformin, the B-A transport was numerically higher than in the A-B direction but failed to reach statistical significance (Elsby et al., 2017).

On the contrary, Horie et al. observed a markedly higher cellular uptake of OCT1 substrates, i.e., tetraethylammonium (TEA), quinidine and metformin, after the apical side cells exposure in comparison with the basolateral side (Horie et al., 2011).

However, this study does not represent a typical bidirectional transport study and possesses a substantial experimental bias. Considering that in vectorial transport studies, Caco-2 cell monolayers are grown on a porous filter membrane (e.g., Transwell^®^ inserts) it becomes clear that the apical membrane is freely accessible to a drug, whereas the basolateral membrane is partly shielded by the artificial filter membrane. Thus, substantial differences in freely assessable membrane are neglected and it remains uncertain whether the substrates may stick to the filter membrane. In each case, polar OCT1 substrates are not expected to diffuse freely across the lipophilic membrane. This limitation would have been canceled out in comparative A-B and B-A transport studies. However, these control experiments have not been performed. The interpretation of those experiments is moreover complicated by the fact that the apical membrane forms brush border membrane-like microvilli resulting in a substantially higher surface area compared to the basolateral membrane which is so far not considered when calculating intestinal effective permeability (Peff) values. Due to these limitations, accumulation studies in Caco-2 cells are not expected to provide valid conclusions on the cellular localization of OCT1. Because Han et al. used the same methodical approach for transport studies of TEA and pentamidine, the derived conclusions on apical OCT1 remain questionable (Han et al., 2013). In this study, a markedly lower uptake of pentamidine and TEA into Caco-2 cells were observed from the basolateral membrane compared to the apical side; the apical absorption was significantly reduced in the presence of quinidine and mitoxantrone (Han et al., 2013).

Taken together, the available data from bidirectional transport studies of OCT1 substrates across Caco-2 cell monolayers provide evidence for basolateral OCT1 cellular localization.

However, it should be noted that the expression of OCT1 in Caco-2 cells seems to be low and highly variable as also described for many other transporters (Hayeshi et al., 2008). While several studies where able to detect OCT1 mRNA expression (Schwarz et al., 2000; Müller et al., 2005; Seithel et al., 2006; Hilgendorf et al., 2007; Hayeshi et al., 2008; Horie et al., 2011; Brück et al., 2017), protein levels could only be verified by few studies (Han et al., 2013; Vaessen et al., 2017), while other targeted-based studies failed to detect OCT1 protein (Uchida et al., 2015; Ölander et al., 2016; Brück et al., 2017).

Given the already mentioned complexity and uncertainties in the expression and functional interplay of intestinal and hepatic OCT1, animal studies in rodents appear to be a promising approach to extrapolate findings to humans. However, general differences between rodents and human should be critically considered, e.g., different expression levels of transporters, differences in blood flow, bile flow and enzymatic activity (Cao et al., 2006; Glaeser and Fromm, 2008).

In rodents, OCT1 was also shown to be strongly expressed in the liver, kidney and small intestine. Here, OCT1 was located in the sinusoidal membrane of hepatocytes, in the basolateral membrane of enterocytes, and the basolateral membrane of epithelial cells of proximal tubules (Meyer-Wentrup et al., 1998; Karbach et al., 2000; Chen et al., 2001). The amino acid identity between the human and mouse/rat OCT1 orthologs is 78%. A very recent study by Meyer et al. has comprehensively demonstrated that the difference of about 22% in amino acid sequence could result in tremendous differences in the intrinsic clearance between human and mouse OCT1 (e.g., 4.7-fold higher for mouse Oct1 in metformin uptake) (Meyer et al., 2020) and thereby highlighted the limited transferability of findings from rodent pharmacokinetic models to humans. Nevertheless, although a direct transfer of data observed in animal studies is not possible, general insights into the expression and function of OCT1 are possible.

In this regard, Shu et al. demonstrated in Oct1-deficient mice that the hepatic uptake of metformin was dramatically reduced which resulted in completely abolished glucose-lowering effects of the drug (Shu et al., 2007). However, metformin concentration-time profiles in blood were not different between wild-type and knockout animals (Shu et al., 2007; Shu et al., 2008) (see also paragraph: “Evidence from clinical studies”). Considering that metformin was orally administered in this study and Oct1-knockout mice did not show any changes in their serum exposure, intestinal OCT1 seems not to be principally involved in metformin absorption, which points to a rather basolateral localization of OCT1 as demonstrated by previous immunostaining analysis (Chen et al., 2001). This assumption is also confirmed by a pharmacokinetic study with OCT1 model substrates TEA and MPP+ (1-methyl-4-phenylpyridinium) in Oct1-knockout mice. After intravenous administration of the probe compounds, not only their hepatic accumulation was reduced (4–6-fold) but also their uptake into the intestinal tissue was nearly halved (Jonker et al., 2001). Comparable findings have been observed for metformin; after i. v. administration of the drug, hepatic accumulation was more than 30 times lower in Oct1-knockout mice than that in wild-type animals, while basolateral uptake from blood into the tissue of duodenum, jejunum and ileum was 3–7-fold lower, which suggests a role of OCT1 in intestinal metformin excretion (Wang et al., 2002). In Oct1/2 double knockout mice, intravenously administered sulpiride resulted in significantly higher serum exposure but lower accumulation of the drug in hepatic, renal and small intestinal tissue (Takano et al., 2017).

Similarly, the hepatic exposure and the duodenal content of sumatriptan, fenoterol, ondansetron, and tropisetron after their intravenous administration was lower in Oct1-knockout mice than that in their wild-type counterparts (Morse et al., 2020). Furthermore, this study compared the pharmacokinetics of the above-mentioned drugs after oral and intravenous administration in wild-type and Oct1-knockout mice. After oral administration, maximum serum levels (C_max_) and serum AUC of all investigated drugs were found to be markedly elevated in OCT1-knockout animals; oral bioavailability was not different or even increased. Assuming OCT1 as apically localized and therefore acting as an intestinal uptake transporter, one would expect a significantly decreased oral bioavailability in knockout mice. The study indicates that OCT1 is rather involved in transport from blood into deeper compartments than in uptake from intestinal lumen to blood. Consequently, OCT1 deficiency in knockout mice was associated with increased serum exposure (decreased serum clearance) and with a decreased volume of distribution of the respective substrates. This again suggests that OCT1 is most likely expressed in the basolateral membrane of the enterocytes.

In contrast to this, again the already mentioned study by Han et al., 2013 hypothesized an apical localization of OCT1 as concluded from uptake studies into mouse intestine. However, very similar to the discussed Caco-2 experiments, no vectorial transport study was performed but a rather simple accumulation experiment after either apical or basolateral exposure to the OCT1 substrate pentamidine (Han et al., 2013). While the uptake from the basolateral membrane tended to be higher compared to the apical membrane, only the uptake from the apical side was reduced in the presence of quinidine and desipramine, both inhibitors of OCT1.

Ideally, confirmative transport studies should be performed as bidirectional transport studies using animal or human tissue mounted in the Ussing chamber, which represents so far the gold standard experiment to provide reliable and biorelevant information on the intestinal drug metabolism and transport (Kisser et al., 2017). Although the mentioned method would be suitable to provide further insights into the localization of OCT1, it was so far exclusively used for absorptive studies. However, reliable conclusions can only be derived from bidirectional transport studies (A-B vs. B-A) (Kim et al., 2005; Arnold and Kalia, 2020).

## Evidence from clinical studies

Investigative approaches to estimate the function of intestinal OCT1 comprise pharmacogenetic studies and DDI studies with orally administered OCT1 substrates. The evidence from clinical studies showing that OCT1 might be a clinically relevant intestinal drugs uptake carrier is limited. This can be attributed to the following aspects which counteract reliable conclusions on the distinct role of OCT1 in the human intestine: first, substrates of OCT1 are partly subjected to extensive metabolism (e.g., morphine, codeine, sumatriptan, tramadol); second, multiple other transporters can be involved in the pharmacokinetics of a certain OCT1 substrate (e.g., OCT2, MATE1/2K, P-gp); third, OCT1 is not inducible by prototypical inducers of enzymes and transporters such as rifampin which disqualifies meaningful inductive studies; fourth, likewise, there is a lack of specific inhibitors that can be used *in vivo*; and fifth, the expression and function of intestinal and hepatic OCT1 results in opposite clinical effects in case of transporter inhibition. Hence, inhibition of intestinal OCT1 (assuming its apical localization) is expected to result in decreased oral drug absorption and systemic exposure, whereas inhibition of hepatic uptake will cause increased plasma levels of OCT1 substrates. Thus, a substantial overlap is expected which may mask the certain effect of intestinal OCT1. In the case of intestinal OCT1 on the basolateral membrane of the enterocyte, transporter inhibition may cause to some extent additionally increased serum levels caused by reduced direct intestinal excretion of the drug.

In addition, renal OCT2 and MATE1/2 K may contribute also to clinical DDI studies because they accept many OCT1 substrates (Koepsell et al., 2007; Koepsell, 2015; Koepsell, 2020), i.e., inhibition of renal cation transporters will result in increased drug exposure as seen for inhibition of hepatic uptake by OCT1.

Considering that there are no specific clinical inhibitors of OCT1 available, the use of pharmacogenetic studies in carriers of OCT1 null alleles (∼9% in Caucasians) is expected to provide additional evidence (Zamek-Gliszczynski et al., 2018).

A prominent example of the difficulties in the interpretation of human clinical studies on OCT1 is the antidiabetic drug metformin. In this regard, the first pharmacogenetic study in a small number of healthy volunteers (*N* = 20) indicated that OCT1 significantly affected the serum exposure and efficacy of metformin. In detail, carriers of SCL22A1 loss-of-function alleles showed a reduced response to the drug (Shu et al., 2007) which was explained by the decreased uptake of metformin to its predominate site of action, namely the liver, as concluded from the significantly elevated serum exposure of metformin in carriers of the genetic variants (Shu et al., 2008). However, the extent of increase in metformin serum AUC was rather little (∼20%). A subsequent and more comprehensive pharmacogenetic study in 103 healthy males could not find any significant changes in the serum pharmacokinetics between carriers of the SLC22A1 wild-type or loss-of-function alleles (Tzvetkov et al., 2009). On the contrary, it was found that the renal clearance of metformin was significantly affected by the number of low-activity OCT1 alleles. Thus, the authors concluded that renal OCT1 might be an important carrier in renal elimination of the drug. Although the authors confirmed their hypothesis by providing immunohistochemical staining of human kidneys, which demonstrated OCT1 expression in the luminal (apical) membrane of proximal and distal tubules, more recent targeted proteomics failed to detect substantial levels renal OCT1 (Prasad et al., 2016; Cheung et al., 2019; Oswald et al., 2019).

Finally, Cho et al. observed that the unspecific OCT inhibitor verapamil did not change the serum pharmacokinetics but significantly decreased the glucose-lowering effect of metformin in 12 healthy volunteers (Cho et al., 2014). This finding was also confirmed by an independent group (Christensen et al., 2015). Consequently, one can summarize that the serum pharmacokinetics of metformin is not significantly affected by OCT1 because this frequently prescribed antidiabetic drug is almost exclusively eliminated via the kidney, which does not express OCT1 but OCT2/3 and MATE1/2K (Shu et al., 2008; Tzvetkov et al., 2009; Cho et al., 2014; Zamek-Gliszczynski et al., 2018). In line with this conclusion, metformin was also shown to be accepted by other cation transporters including OCT2, MATE1, and MATE2-K, which contribute to the pharmacokinetics and DDI studies of the drug (Wang et al., 2008; Kusuhara et al., 2011; Ito et al., 2012; Yoon et al., 2013; Cho et al., 2014; Dujic et al., 2015). Accordingly, inhibition of OCT1-mediated hepatic uptake by co-administered drugs are expected to reduce hepatic drug levels and in turn its antihyperglycemic effects with no considerable changes in systemic metformin concentrations (Cho et al., 2014; Sundelin et al., 2017). In the same manner, the observed impact of genetic polymorphisms of SLC22A1 gene on antidiabetic effects of metformin could be explained by differences in tissue exposure to the drug (Shikata et al., 2007; Shu et al., 2007; Cho et al., 2014), which seems to be also affected by sinusoidal efflux transporters (Zamek-Gliszczynski et al., 2013). However, it should be noted that this finding could not be verified in a larger cohort study in 3,450 type 2 diabetes patients on the level of glycated hemoglobin (HbA1c) (Zhou et al., 2009). Moreover, the impact of genetic variants of OCT1 on the metformin response were shown to be population specific (Mofo Mato et al., 2018). This complex example nicely demonstrates that it can be challenging to conclude from pharmacokinetic data alone on the distinct relevance of OCT1. Taken together, DDI studies with metformin should include a pharmacodynamic measure but the drug may not be a suitable drug to conclude on the function of intestinal OCT1 (Zamek-Gliszczynski et al., 2018).

In contrast to metformin, it was demonstrated for several other OCT1 substrates, that OCT1-mediated hepatic uptake is the rate-determining step in their hepatic processing, and thus are expected to be more suitable markers to provide deeper insights into the role of OCT1 for systemic drug exposure; i.e., being probe substrates for clinical DDI studies. An example is the beta2-adrenergic receptor agonist fenoterol, a narrow therapeutic index drug, for which it was demonstrated that SLC22A1 homozygous carriers of loss-of-function alleles possessed about 2-fold higher systemic drug exposure at significantly increased heart rate and blood glucose but significantly lowered serum potassium levels, all of which are pharmacodynamic side effects of the drug (Tzvetkov et al., 2018). However, fenoterol was administered in this study via intravenous infusion, which hampers conclusions on intestinal OCT1 function. Considering furthermore, that fenoterol is regularly administered via inhalation for the treatment of asthma and COPD, it is not surprising that human DDI studies on OCT1 are, unfortunately, not available.

Another even more frequently used example is the opioid analgetic drug tramadol, which active metabolite O-desmethyl tramadol is a substrate of OCT1. Similarily to fenoterol, oral administration of tramadol resulted in about 2-fold greater metabolite exposure in healthy volunteers carrying loss-of-function SLC22A1 polymorphisms, resulting in significantly prolonged miosis, i.e., a characteristic opioid effect (Tzvetkov et al., 2011). Moreover, these prolonged opioid effects resulted in decreased self-administration of the drug in patients suffering from postoperative pain in clinical practice (Stamer et al., 2016). As a considerable limitation, tramadol undergoes extensive metabolism by CYP2D6 which represents a substantial confounder in DDIs studies on OCT1. An example for this aspect might be the observed decrease of the analgesic efficacy of tramadol in the presence of ondansetron (De Witte et al., 2001; Vale et al., 2011). As both drugs are substrates of CYP2D6 and OCT1, the distinct contribution of OCT1 remains uncertain (Tzvetkov et al., 2012). Thus, the function of intestinal OCT1 can not be directly anticipated from DDI studies with tramadol because of interferences of the perpetrator drug with the hepatic oxidative metabolism.

Under consideration of the first examples and the respective limitations, an OCT1 substrate which might be suitable to provide further insights into the expression and function of intestinal OCT1 requires the following features: first, oral administration (oral dosage form available); second, no or only minor metabolism; third, no or only minor passive diffusion; and fourth, no other transporters influencing its pharmacokinetics in a significant manner.

Applying these criteria to the substrates summarized in Table 1, they would disqualify at first glance acyclovir, codeine, diphenhydramine, formoterol, fluoxetine, ipratropium, ketamine, morphine, oxaliplatin, oxybutynin, procainamide, proguanil, salbutamol, terazosin, tiotropium, triamterene, and trimethoprim. On the other side, drugs such as amantadine, amiloride, amisulpride, atenolol, butylscopolamine, etilefrine, ranitidine, sulpiride, sumatriptan, and trospium may be suitable to derive conclusions on intestinal OCT1.

Because potent inducers of OCT1 are not available, only pharmacogenetic and DDI studies with orally administered unspecific inhibitors of OCT transporters can be used to provide further insights into intestinal OCT1. Table 3 summarizes appropriate inhibitors that are expected to be suitable candidates in clinical studies. As discussed elsewhere, there is no doubt that there is a tremendous variability in the published IC50 values even when using the same probe substrate (e.g., MPP+), which makes it challenging to estimate clinically relevant DDIs (Nies et al., 2011; Koepsell, 2015). This uncertainty is further amplified by the partly unknown concentrations *in vivo*; e.g., in portal vein (up to 100-fold higher compared to the systemic blood concentration) relevant for OCT1-mediated uptake into the liver or in the intestinal lumen affecting interaction with intestinal uptake carriers (assumption so far: dose/250 ml, although the intestine is known to contain much less volume of water (50–100 ml) (Schiller et al., 2005)). However, only for very few of the mentioned OCT1 substrates, confirmative clinical pharmacogenetic or DDI studies have been performed.

**TABLE 3:** Overview of clinically relevant drugs that are orally administered and potent inhibitors of OCT1.

Drug/compound	Class	Inhibitory effect	References
Amitriptyline	Non-selective NSRI	IC_50_ = 4.4 µM	Tzvetkov et al. (2013)
Cimetidine	H_2_-receptor antagonist	IC_50_ = 60 µM	Koepsell (2020)
Citalopram	SSRI	IC_50_ = 2.8 µM	Koepsell et al. (2007)
Clonidine	α-adrenoceptor antagonist	IC_50_ = 0.6–6.5 µM	Koepsell et al. (2007)
Desipramine	Non-selective NSRI	IC_50_ = 5.4 µM	Koepsell et al. (2007)
Diphenhydramine	H_1_-receptor antagonist	IC_50_ = 3.4 µM	Müller et al. (2005)
Fluoxetine	SSRI	IC_50_ = 6.0 µM	Tzvetkov et al. (2013)
Imipramine	Non-selective NSRI	IC_50_ = 6.2 µM	Tzvetkov et al. (2013)
Memantine	NMDA receptor antagonist	IC_50_ = 3.7 µM	Busch et al. (1998)
Metoclopramide	D_2_/5-HT_3_ receptor anatgonist	IC_50_ = 16–95 µM	Koepsell (2020)
Morphine	Opioid receptor agonist	IC_50_ = 4.2–28 µM	Koepsell (2020)
Ondansetron	5-HT_3_ receptor antagonist	IC_50_ = 1.2 µM	Tzvetkov et al. (2013)
Oxybutynin	Muscarinic receptor antagonist	IC_50_ = 20 µM	Koepsell (2020)
Prazosin	α-adrenoceptor antagonist	IC_50_ = 1.8 µM	Hayer-Zillgen et al. (2002)
Quinidine	Na^+^channel blocker (antiarrhythmic)	IC_50_ = 18 µM	Koepsell et al. (2007)
Quinine	Antimalaria drug	IC_50_ = 13–23 µM	Koepsell et al. (2007)
Ranitidine	H_1_-receptor antagonist	IC_50_ = 28 µM	Müller et al. (2005)
Ritonavir	HIV protease inhibitor	IC_50_ = 5.2 µM	Zhang et al. (2000)
Trospium	Muscarinic receptor antagonist	IC_50_ = 5.3–18 µM	Koepsell (2020)
Verapamil	Ca^2+^channel blocker	IC_50_ = 1.6–2.9 µM	Koepsell et al. (2007), Tzvetkov et al. (2013)

IC_50_, half maximal inhibitory concentration; NMDA, N-methyl-D-aspartate; NSRI, norepinephrine and serotonin reuptake inhibitor; SSRI, selective serotonin reuptake inhibitor.

A well investigated drug in this regard is the antimigraine drug sumatriptan, which systemic exposure was over 2-fold increased after oral administration in carriers of SLC22A1 loss-of-function alleles (Matthaei et al., 2016). Although sumatriptan is subjected to extensive first pass metabolism (bioavailability, ∼15%) by monoamine oxidase A (MAO-A), this metabolic pathway might be only a confounder in very few DDI studies, because known potent inhibitors are rather less frequently prescribed drugs including moclobemide, tranylcypromine, linezolid, selegiline, and zonisamide. Despite the fact that significant DDIs studies with known unspecific inhibitors of OCTs (Table 3) cannot be found, the described pharmacogenetic data on sumatriptan do not support the hypothesis of apically expressed OCT1 in the human intestine. Otherwise, carriers of loss-of-function alleles should exhibit lower instead of higher drug exposure as observed by Matthaei and colleagues (Matthaei et al., 2016).

Similarily to sumatriptan, morphine is also in most cases orally administered and was shown to be a substrate of OCT1 (Tzvetkov et al., 2013). However, due to its pronounced lipophilicity (logP, 0.9) and its moderate basicity (pKa, 8.2), considerable diffusion from the systemic circulation can be assumed (ionization degree at pH 7.4, 86.3%), which may counteract reliable conclusions on the quantitative contribution of hepatic OCT1. Moreover, extensive glucuronidation via UGT2B7, which is predominately expressed in the human liver (Drozdzik et al., 2018), further limits application of morphine as an OCT1 probe drug. In contrast to this, in the intestinal lumen (pH 3–5), over 99.99% of morphine is expected to be ionized and would necessarily require an uptake transporter such as OCT1. From this perspective, oral morphine might be a suitable drug to derive conclusion on intestinal OCT1. Associated to this, Nielson et al. could not find any changes in the pharmacokinetics or pharmacodynamic effects of orally administered morphine in 37 healthy volunteers related to common genetic variants of SLC22A1, ABCB1, and UGT2B7 (Nielsen et al., 2017). In line with those findings, there are no DDI studies with orally administered morphine and the aforementioned inhibitors of OCT1 (Table 3) compromising the oral opioid absorption. In contrast to this, neither Cmax nor serum AUC of oral controlled release morphine were significantly different in combination with oral metoclopramide (MCP). Only morphine tmax occurred significantly earlier in the MCP group as explained by the known prokinetic effect of MCP resulting in accelerated gastric emptying (Manara et al., 1988). The simultaneous oral intake of morphine and the antiarrhythmic drug quinidine resulted even in a 1.9-fold and 1.6-fold higher C_max_ and AUC of morphine and significantly increased opioid effects (Kharasch et al., 2003). A similar outcome has been reported for the oral combination of morphine and ranitidine. Here, AUC0-90min of morphine was 1.5-fold increased in the presence of ranitidine (Aasmundstad and Størset, 1998). While those effects can be attributed to inhibition of intestinal P-gp (morphine is a P-gp substrate, while quinidine and ranitidine are inhibitors of OCT1 and P-gp), one can conclude from the pharmacogenetic and DDI studies again that OCT1 may not be localized in the apical membrane of the human enterocytes.

Interestingly, significant associations between SLC22A1 loss-of-function variants and the pharmacokinetics of morphine (i.e., morphine clearance was significantly reduced) and higher frequency of side effects have been observed in children after intravenous administration (Fukuda et al., 2013; Venkatasubramanian et al., 2014; Balyan et al., 2017; Hahn et al., 2019). Furthermore, Tzvetkov et al. found gene dose-dependent changes in the pharmacokinetics of morphine in healthy volunteers after oral administration of the prodrug codeine, which is bioactivated in the liver via CYP2D6 to morphine (Tzvetkov et al., 2013; Drozdzik et al., 2018). However, although those studies demonstrated that the pharmacokinetics of morphine is significantly affected by OCT1 (despite the aforementioned limitations), they did not allow any conclusions on the function of intestinal OCT1, since morphine was in both scenarios administered to the systemic circulation, either directly by intravenous administration or indirectly by using a prodrug, which has to be bioactivated in the liver.

Additional arguments against OCT1 at the apical membrane in the human intestine provide interaction studies of atenolol with cimetidine, metoclopramide with ranitidine and metformin with trospium (Houtzagers et al., 1982; Leucuţa et al., 2004; Oefelein et al., 2013). In all studies, serum levels of the victim drugs were not changed or only marginally elevated (MCP). However, DDI studies with cimetidine have to be interpreted with caution as this drug inhibits also the renal secretion of many drugs in proximal tubules by blocking OCT2-mediated uptake at the basolateral membrane and/or inhibition of efflux at the apical membrane mediated by MATE1, MATE2-K, OCTN1, and/or OCTN2 (Koepsell, 2020).

Finally, the poorly absorbable bladder spasmolytic trospium (intestinal absorption and oral bioavailability about 10%) might be a good candidate to conclude on the function of intestinal OCT1 because this drug is given orally, is not metabolized and is not subjected to significant hepatic uptake but undergoes almost exclusively renal excretion (Doroshyenko et al., 2005). In this regard, interaction studies with oral ranitidine and metformin are available (Oefelein et al., 2013; Abebe et al., 2020). In both studies, trospium serum AUC and Cmax were not significantly different in the presence of the inhibitor of OCT1. In the interaction study with metformin, the systemic exposure of trospium was numerically even slightly increased. Also these studies indicate that OCT1 might not be present in the apical but rather in the basolateral membrane of the human enterocytes.

**FIGURE 3:**
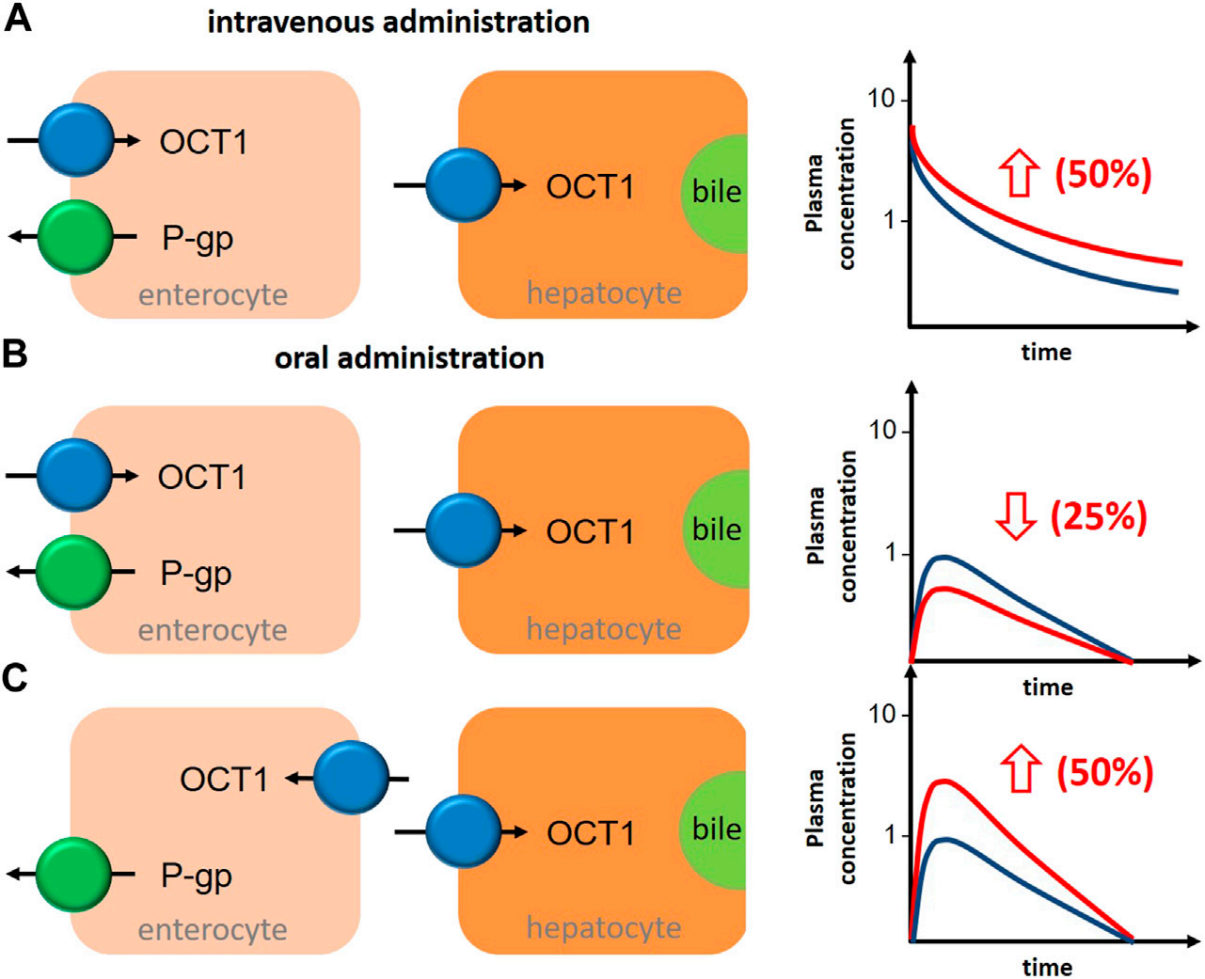
Schematic illustration about the impact of OCT1 inhibition in human liver and intestine on the bioavailability of the victim drug in depencence on the localization of intestinal OCT1 and the route of drug administration. **(A)**, after intravenous administration of an OCT1 substrate, inhibition of hepatic OCT1 will increase systemic drug exposure by 50%. **(B)**, after oral administration of an OCT1 substrate, inhibition of intestinal (apical localization) and hepatic OCT1 will decrease oral bioavailability by 25%. **(C)**, after oral administration of an OCT1 substrate, inhibition of intestinal (basolateral localization) and hepatic OCT1 will increase oral bioavailability by 50%. General assumptions for all estimations: intestinal and hepatic uptake of the drug are 50% and mediated by OCT1; OCT1 inhibition results in 50% reduction in the intestinal absorption (fa) and/or hepatic uptake (blue graph, OCT1 substrate without inhibitor; red graph, OCT1 substrate with inhibitor).

However, as mentioned earlier, the interpretation of clinical interaction studies with OCT1 substrates and inhibitors as summarized in Table 4 is complicated by the interference of intestinal and hepatic uptake function of OCT1 resulting theoretically in opposite effects–assuming OCT1 in the apical membrane of the enterocytes contributing significantly to oral drug absorption (Figure 3).

**TABLE 4:** Overview of clinical drug-drug interactions which may allow conclusions on intestinal OCT1.

Substrate (victim drug)	Perpetrator (inhibitor)	PK change	References
Atenolol (100 mg, oral)	Cimetidine (1,000 mg, oral)	AUC and cmax unchanged	Houtzagers et al. (1982)
Metformin (500 mg, BID, oral)	Trospium (60 mg, QID, oral)	AUC and cmax unchanged	Oefelein et al. (2013)
Metoclopramide (20 mg, oral)	Ranitidine (150 mg, oral)	AUC↑, +13% (*p* < 0.05); Cmax↑, +12% (N.S.)	Leucuţa et al. (2004)
Morphine (20 mg, oral)	Metoclopramide (10 mg, oral)	AUC and cmax unchanged	Manara et al. (1988)
Morphine (30 mg, oral)	Quinidine (600 mg, oral)	AUC↑, 1.6-fold; Cmax↑, 1.9-fold	Kharasch et al. (2003)
Morphine (10 mg, oral)	Ranitidine (150 mg, oral)	AUC_0–90 min_, ↑1.5-fold	Aasmundstad und Størset (1998)
Trospium (60 mg, QID, oral)	Metformin (500 mg, BID, oral)	AUC↑, +29% (N.S.); Cmax↑, +34% (N.S.)	Oefelein et al. (2013)
Trospium (30 mg, oral)	Ranitidine (300 mg, oral)	AUC and cmax unchanged	Abebe et al.(2020)

AUC, area under the concentration-time curve; BID, twice daily; CL, clearance; Cmax, maximum serum concentration; Css, trough serum concentrations at steady-state; d, days; MD, multiple doses; QID, four times daily; SID, once daily; SD, single dose; t1/2, elimination half-life.

Assuming oversimplified that intestinal and hepatic uptake would contribute equally to the bioavailability of a certain OCT1 substrate, simultaneous inhibition of intestinal and hepatic OCT1 would result in only slightly changed systemic exposure of the victim drug (Figure 3B). However, this is mostly not the case as the intestinal absorption of most OCT1 substrates is limited (suggesting a rate-determining intestinal transporter) and their hepatic extraction and biliary excretion is even lower (predominate renal excretion of about 80–90%). Based on the simple equation on oral bioavailability F = fa*fg*fh, where (fa) is the absorbed dose fraction (fg) is the fraction of drug escaping first-pass gut wall metabolism that enters the portal blood, and (fh) is the fraction of drug escaping hepatic metabolism and biliary secretion entering the systemic circulation (Huang et al., 2009), and assuming that (fg) is not relevant for a confirmative OCT1 probe drug (fg = 1), systemic drug exposure is a function of intestinal absorption and hepatic extraction. Applying this very simple conception to the discussed interference of intestinal and hepatic OCT1 transport, it becomes clear that the contribution of intestinal OCT1 (assuming its apical localization) is expected to dominate the entire process (Table 5); i.e., interaction studies with OCT1 inhibitors should result in pronounced reduction of serum exposure to OCT1 substrates. As none of the available studies showed this result, there are no arguments from pharmacogenetic and DDI studies to assume an apical localization of OCT1 in the human enterocytes but rather its presence in the opposite membrane. As mentioned earlies, this oversimplification omits the potential simultaneous inhibition of renal OCT2/3 and MATE1/2K transporters by unspecific inhibitors of OCT1.

**TABLE 5:** Estimated impact on oral bioavailability (F) of OCT1 substrates caused by inhibition of intestinal and/or hepatic OCT1 and observed clinical data.

Scenario	Atenolol	Metoclopramide	Metformin	Trospium
No inhibition of intestinal and hepatic OCT1	0.425	0.714	0.48	0.095
Predicted inhibition of intestinal OCT1 (assuming apical localization)	0.213 (↓50%)	0.357 (↓50%)	0.24 (↓50%)	0.048 (↓50%)
Predicted inhibition of hepatic OCT1 only (i.v. administration or basolateral intestinal OCT1)	0.463 (↑9%)	0.777 (↑9%)	0.54 (↑13%)	0.098 (↑3%)
Predicted inhibition of intestinal (apical) and hepatic OCT1 (oral administration)	0.231 (↓46%)	0.389 (↓46%)	0.27 (↓44%)	0.049 (↓49%)
Observed interaction data	unchanged AUC Houtzagers et al. (1982)	AUC↑, 13% Leucuţa et al. (2004)	unchanged AUC Oefelein et al. (2013)	unchanged AUC Abebe et al. (2020)

Used data for estimations: Atenolol (fa, 0.5; fh, 0.85), Metoclopramide (fa, 0.84; fh, 0.85); Metformin (fa, 0.6; fh, 0.8) and Trospium (fa, 0.1; fh, 0.95). In the case of inhibition, 50% reduction of intestinal absorption or hepatic extraction was assumed. As data on fh were not available, they have been indirectly estimated from excretion pathways (fh ∼ renal excretion after i.v. administration).

## Summary and Conclusion

There is no doubt that hepatic OCT1 can influence the pharmacokinetics and in turn the efficacy and safety of several drugs in a significant manner (Jonker and Schinkel, 2004; Koepsell et al., 2007; Shu et al., 2007; Koepsell, 2015, 2020). In this regard, genetic polymorphisms and DDIs were shown to result in drastically changed serum levels of the respective substrates. Consequently, the latest update of the International Transporter Consortium emphasized OCT1 as a transporter of emerging clinical importance (Zamek-Gliszczynski et al., 2018).

As OCT1 was also shown to be expressed in the human intestine, it was assumed to be involved in the intestinal absorption of drugs. Despite its unequivocal intestinal abundance, the distinct localization in the enterocytes still remains uncertain as two independent studies identified OCT1 either in the apical or the basolateral membrane (Müller et al., 2005; Han et al., 2013). However, only if OCT1 is present in the apical membrane facing the intestinal lumen it can contribute directly to oral drug absorption. There was recently a similar discussion on the localization of OATP2B1 in the human intestine. Targeted proteomics analysis of the intestinal membranes along with functional studies in Caco-2 cells and intestinal tissue from animals and human clarified OATP2B1 as a basolateral carrier (Keiser et al., 2017) and ruled it out to be a transporter involved in intestinal drug absorption. Very recent studies from knockout mice indicate that Oatp2b1 might be involved in intestinal drug absorption (Medwid et al., 2019; Chen et al., 2020). However, considering that human OATP2B1and mice Oatp2b1share only 74.6% amino acid homology, additional transporters are involved in the pharmacokinetics of the investigated drugs (fexofenadine, rosuvastatin and fluvastatin) and that general limitations on the direct comparison of human and rodent pharmacokinetics exist, these findings must be interpreted with caution. Further studies with human intestinal tissue are required, which is also true for OCT1.

Accordingly, most bidirectional transport studies of OCT1 substrates across Caco-2 cells demonstrated a markedly higher secretory transport compared to the opposite direction (B-A > A-B), which suggest a basolateral localization of OCT1 (Lee et al., 2002; Watanabe et al., 2002; Kuwayama et al., 2008). As recently shown, OCT1 also contributes to thiamine uptake (Chen et al., 2014). Here, Oct1 knockout in mice was associated with dramatically reduced uptake of intravenously administered thiamine into intestinal tissues confirming a basolateral localization of OCT1. This assumption is also supported by several other former animal experiments, in which direct excretion of intravenously administered OCT1 substrates into the intestinal lumen was shown to be markedly lower in Oct1-knockout mice (Jonker et al., 2001; Wang et al., 2002; Takano et al., 2017). Moreover, oral administration of OCT1 substrates resulted in unchanged or even substantially increased serum levels in Oct1-knockout mice (Morse et al., 2020). These data are in line with the basolateral localization of Oct1 in the murine intestine as observed by immunohistochemistry (Chen et al., 2001). Considering also the basolateral (sinusoidal) localization of OCT1 in hepatocytes and the fact that most transporters show the same localization in liver, kidney and intestine (e.g., P-gp, MRP2, MRP3, BCRP, MATE1) it appears reasonable to assume OCT1 as a basolateral transporter in human gut. Interestingly, OCT1 was also speculated to be involved in the efflux of acylcarnitines from the liver to the systemic circulation (Kim et al., 2017). Assuming OCT1 as a bidirectional transporter, it seems possible that it may also be involved in drug absorption on the basolateral membrane of the enterocytes. However, this hypothesis needs to be proven by additional studies.

Finally, also the available pharmacogenetic and DDI studies do not provide evidence for apically localized intestinal OCT1. However, the interpretation of clinical studies is complicated considering the complex contribution of intestinal, hepatic, and renal cation transporters. Moreover, confirmative induction studies as regularly performed for P-gp or cytochrome P450 enzymes are not possible for OCT1.

In conclusion, available evidence from expression studies, *in vitro* and animal experiments as well as data from clinical studies suggest that OCT1 is localized in the basolateral membrane of the enterocytes and cannot be considered as an uptake transporter in the human intestine.

Basolateral OCT1 in the enterocytes would imply its involvement in the intestinal excretion of drug from the systemic circulation. For this secretory net transport across the enterocytes, P-gp can be expected a relevant efflux transporter in the apical membrane because it accepts many OCT1 substrates. Indeed, this intestinal elimination pathway has been observed in several animal studies after intravenous administration of OCT1 substrates (Suttle and Brouwer, 1995; Jonker et al., 2001; Wang et al., 2002; Takano et al., 2017) but also in clinical pilot studies demonstrating direct intestinal secretion of supiride and ranitidine, both substrates of OCT1 and P-gp (Gramatté et al., 1994; Takano et al., 2017). Thus, OCT1 should be considered as a basolateral uptake carrier contributing to the intestinal elimination of cationic compounds from the systemic circulation. However, considering its rather low protein abundance and its mode of action, the allover pharmacokinetic relevance of this elimination pathway appears to be low.

On the contrary, assuming OCT1 in the apical membrane of the enterocytes (Han et al., 2013) would raise the question on the feasibility of an absorptive net transport across the intestinal epithelia because the enterocytes lack cation transporters in the basolateral membrane allowing a flux out of the intercellular space (Proctor et al., 2016).

Given the assumption that OCT1 is not present in the apical membrane of the human enterocytes, which mechanisms may be involved in the intestinal uptake of cationic compounds? Beside mechanisms of paracellular transport as discussed elsewhere (Proctor et al., 2016), the intestinal brush border membrane also expresses several other transporters that have been shown to be involved in the uptake of cationic compounds such as the plasma membrane monoamine transporter (PMAT), the thiamine transporter 2 (THTR2), the choline transporter 1 (CHT1), the norepinephrine transporter 1 (NET1), the serotonin transporter (SERT), and the dopamine transporter 1 (DAT1).

For an unequivocal proof for the localization of intestinal OCT1, targeted proteomic analysis of apical and basolateral membrane fractions of the human intestinal mucosa and bidirectional transport studies of established non-metabolized OCT1 substrates across human intestinal tissue from a sufficient number of volunteers (e.g., carriers of SLC22A1 loss of function alleles vs. carriers of the wild-type or tissue from wild-type carriers in the absence and presence of OCT1 inhibitors) would be required.
